# Expression of anti-chikungunya single-domain antibodies in transgenic *Aedes aegypti* reduces vector competence for chikungunya virus and Mayaro virus

**DOI:** 10.3389/fmicb.2023.1189176

**Published:** 2023-06-12

**Authors:** Emily M. Webb, Austin Compton, Pallavi Rai, Christina Chuong, Sally L. Paulson, Zhijian Tu, James Weger-Lucarelli

**Affiliations:** ^1^Department of Entomology, Fralin Life Sciences Institute, Virginia Polytechnic Institute and State University, Blacksburg, VA, United States; ^2^Department of Biochemistry, Fralin Life Sciences Institute, Virginia Polytechnic Institute and State University, Blacksburg, VA, United States; ^3^Department of Biomedical Sciences and Pathobiology, VA-MD Regional College of Veterinary Medicine, Virginia Polytechnic Institute and State University, Blacksburg, VA, United States; ^4^Center for Emerging, Zoonotic and Arthropod-Borne Pathogens, Fralin Life Sciences Institute, Virginia Polytechnic Institute and State University, Blacksburg, VA, United States

**Keywords:** alphavirus, chikungunya virus (CHIKV), Mayaro virus (MAYV), single-domain antibodies (sdAb), *Aedes* (*Ae.*) *aegypti*, transgenic mosquitoes

## Abstract

Chikungunya virus (CHIKV) and Mayaro virus (MAYV) are closely related alphaviruses that cause acute febrile illness accompanied by an incapacitating polyarthralgia that can persist for years following initial infection. In conjunction with sporadic outbreaks throughout the sub-tropical regions of the Americas, increased global travel to CHIKV- and MAYV-endemic areas has resulted in imported cases of MAYV, as well as imported cases and autochthonous transmission of CHIKV, within the United States and Europe. With increasing prevalence of CHIKV worldwide and MAYV throughout the Americas within the last decade, a heavy focus has been placed on control and prevention programs. To date, the most effective means of controlling the spread of these viruses is through mosquito control programs. However, current programs have limitations in their effectiveness; therefore, novel approaches are necessary to control the spread of these crippling pathogens and lessen their disease burden. We have previously identified and characterized an anti-CHIKV single-domain antibody (sdAb) that potently neutralizes several alphaviruses including Ross River virus and Mayaro virus. Given the close antigenic relationship between MAYV and CHIKV, we formulated a single defense strategy to combat both emerging arboviruses: we generated transgenic *Aedes aegypti* mosquitoes that express two camelid-derived anti-CHIKV sdAbs. Following an infectious bloodmeal, we observed significant reduction in CHIKV and MAYV replication and transmission potential in sdAb-expressing transgenic compared to wild-type mosquitoes; thus, this strategy provides a novel approach to controlling and preventing outbreaks of these pathogens that reduce quality of life throughout the tropical regions of the world.

## Introduction

The widely distributed *Aedes* spp. mosquitoes, notably *Ae. aegypti* and *Ae. albopictus*, are vectors for various debilitating pathogens such as yellow fever virus (YFV), dengue virus (DENV), Zika virus (ZIKV), Rift Valley fever virus (RVFV), and chikungunya virus (CHIKV) (Powell, 2018). The primary method of reducing the spread of most arboviral diseases is limiting the interaction between mosquitoes and their human hosts. This is mainly achieved by educating the public on the use of personal protective equipment (e.g., long-sleeved clothing, screens and/or netting, etc.), the use of insect repellents, and ridding properties of standing water (e.g., tires, buckets, pools, etc.). While these tools have proven to be beneficial, some mosquito species, particularly *Ae. aegypti*, have evolved to live in close proximity to humans and are extremely anthropophilic rendering these methods inadequately effective. Therefore, insecticides are used to reduce populations of mosquitoes in more urban settings; however, the environmental impacts of these potentially toxic chemicals have led to great concern. Due to this concern, modern development is focused on the production of insecticides that require fewer applications, have increased specificity, and do not bioaccumulate (Reeves et al., 2019). However, the limited number of approved insecticides, the potency of available insecticides, and, most importantly, the development of insecticide resistance has all played a major role in the emergence and/or re-emergence of many arthropod-borne (arboviral) diseases (Corbel et al., 2017).

In order to control mosquito populations and/or impede disease transmission, novel strategies including genetic manipulations of mosquitoes have been explored over the last few decades. Recent advances have resulted in the release of genetically modified mosquitoes that carry lethal genes in an attempt to suppress natural mosquito populations and, thus, minimize their interaction with human hosts (i.e., population suppression) (Harris et al., 2012; Carvalho et al., 2015; Waltz, 2021; Wang et al., 2021). Alternatively, scientists have developed control programs that focus on replacing natural disease-carrying mosquito populations with pathogen-refractory mosquito populations (i.e., population modification) (Walker et al., 2011; Aliota et al., 2016; Yakob et al., 2017). While both population suppression and population modification strategies are promising, there are several concerns regarding what the impact of reducing mosquito populations may have on the environment (Fang, 2010) as well as concerns regarding the stability of transinfected organisms, such as *Wolbachia*, within certain environmental conditions (e.g., climate and mosquito diet) (Ross et al., 2019). With this being said, there is still a crucial need for the continued exploration of novel mosquito control strategies in order to combat arboviruses.

**Figure 1:**
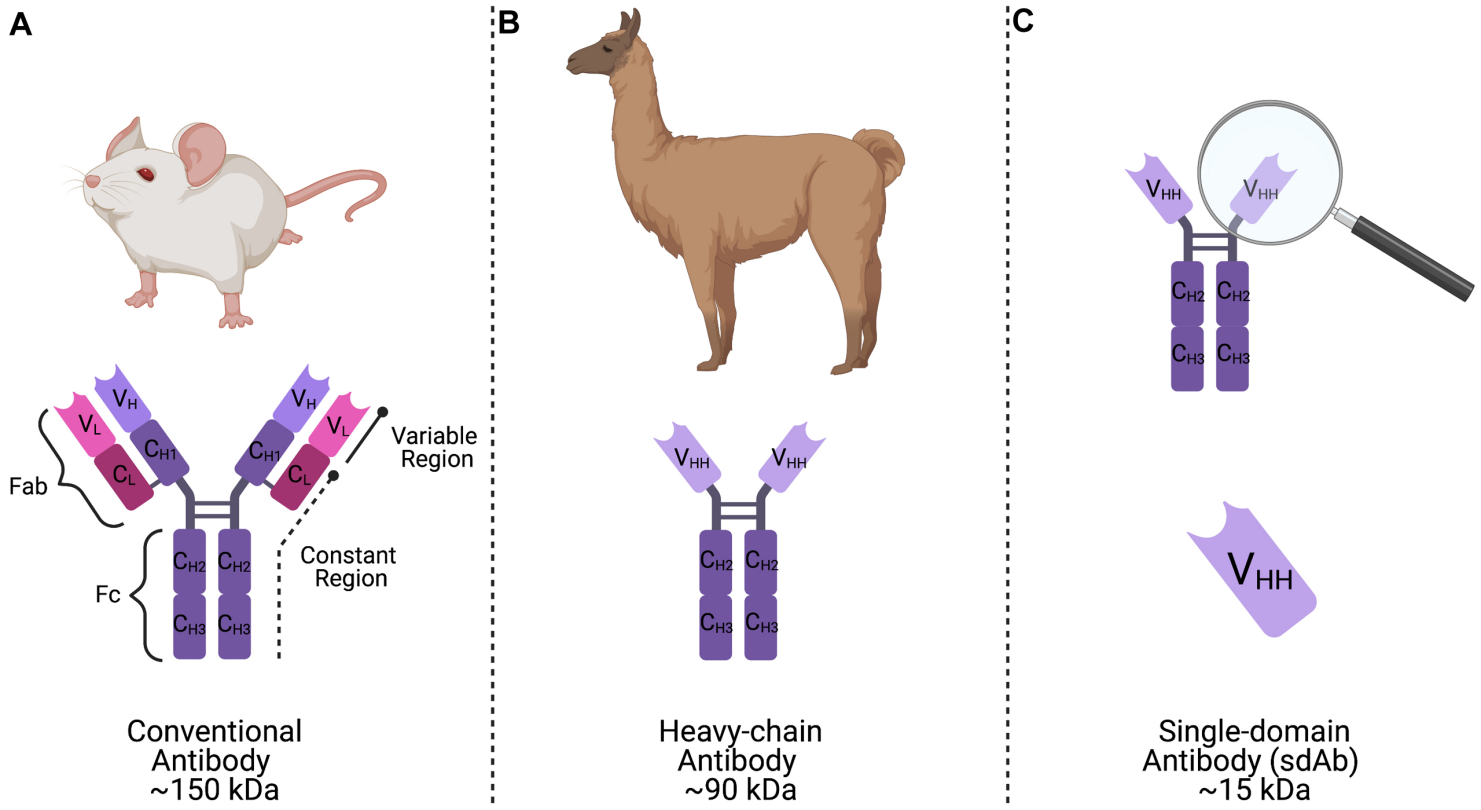
Representation of conventional, heavy-chain, and single-domain antibodies. **(A)** Conventional mammalian antibodies are comprised of a basic structure consisting of two variable and constant heavy chains and two variable and constant light chains forming the antigen binding fragment (Fab) region, as well as two constant heavy chains forming the crystallizable fragment (Fc) region. **(B)** Camelid heavy-chain antibodies are composed of two identical constant heavy chains and a variable region (VHH) linked by a curved hinge that is responsible for antigen-binding activity. **(C)** A single-domain antibody (sdAb) is the single antigen binding domain of camelid heavy-chain antibodies, or the VHH region, that has been synthetically produced and retains full functionality.

CHIKV and Mayaro virus (MAYV) are medically-relevant arboviruses belonging to the *Togaviridae* virus family and *Alphavirus* genus that infect millions of people annually, with CHIKV being responsible for the vast majority of these cases. Infections with these viruses typically result in a febrile illness that is accompanied by incapacitating joint pain that can last for years following infection (Partidos et al., 2011; Elsinga et al., 2017). Both CHIKV and MAYV have become increasingly prevalent throughout the Americas within the past several years. Thus, there is an expanding need for novel approaches to control these viruses. Several studies have been conducted to understand cross-protective immunity among alphaviruses (Hearn, 1961; Latif et al., 1979; Peck et al., 1979; Wust et al., 1987; Partidos et al., 2012; Fox et al., 2015; Webb et al., 2019). In particular, antibodies developed against CHIKV can neutralize and cross-protect against infection with MAYV (Fox et al., 2015; Webb et al., 2019). Our previous studies have characterized a camelid-derived anti-CHIKV single-domain antibody (sdAb) that has potent cross-protective activity against other medically-relevant alphaviruses, including MAYV (Liu et al., 2021). Camelid sdAbs have many advantages when compared to conventional mammalian antibodies, including: increased thermostability, ease of production, and small size (~15 kDa) when compared to conventional mammalian antibodies (Figure 1).

Ultimately, these advantages and the cross-protective capabilities of the aforementioned CHIKV sdAbs provide a unique antiviral strategy. First, we demonstrated potent neutralization of CHIKV and MAYV in the presence of these two anti-CHIKV sdAbs *in vitro*. With these data in mind, we then designed and developed transgenic *Ae*. *aegypti* mosquitoes that express two different anti-CHIKV sdAbs targeting both CHIKV and MAYV. We then observed a statistically significant reduction of CHIKV and MAYV dissemination and transmission rates in the sdAb-expressing transgenic mosquitoes and reduced viral titers throughout the transgenic mosquitoes, thus limiting the overall transmission potential of both viruses. This transgenic strategy provides a proof of concept for the use of camelid- derived sdAbs in modified mosquito populations and has the potential to be used in future population modification strategies to co-target CHIKV, MAYV, and possibly other closely-related alphaviruses.

## Methods and materials

### Virus propagation and cell culture

The CHIKV and MAYV strains used in this study were obtained from the World Reference Center for Emerging Viruses and Arboviruses (WRCEVA) at the University of Texas Medical Branch (Galveston, TX), with the exception CHIKV SL-15649 (ECSA lineage), a kind gift from Dr. Mark Heise (Morrison et al., 2011). The viruses obtained from WRCEVA include MAYV 12A (genotype L), MAYV TRVL-4675 (genotype D), and CHIKV H-20235 (Asian lineage). African green monkey kidney (Vero) cells were used to propagate viruses. Vero cells were purchased from American Type Culture Collection (Manassas, VA; CCL-81) and maintained with Dulbecco’s Modified Eagle Medium (DMEM; Sigma Aldrich; St. Louis, MO) supplemented with L-glutamine, 5% fetal bovine serum (FBS), and 1% gentamycin (herein referred to as Vero cells maintenance media) and kept at 37°C with 5% CO_2_. Vero cells were grown to ~85% confluency before being infected with the respective viruses at an MOI of 0.01 in viral diluent (RPMI-1640 media with 25 mM HEPES, 1% BSA, 50 μg/mL Gentamicin, 2.5 μg/mL Amphotericin B). The cells were infected for 1 h at 37°C, with rocking every 10–15 min. After 1 h of incubation, Vero cell maintenance media was added to the flask. Once 50–75% of cells demonstrated cytopathic effects (CPE), the cellular supernatant was collected and clarified by centrifugation at 4°C before storage at −80°C.

### Plaque assays

Virus stocks were titrated by plaque assay before use in subsequent experiments. Serial 10-fold dilutions of each sample were made in viral diluent and then added to confluent monolayers of Vero cells. After a 1 h incubation period, an overlay containing 1.5% methylcellulose was added. Plaques were visualized following formalin fixation and staining with crystal violet 3 days post-infection.

### Plaque reduction neutralization tests

Purified sdAb samples were serially diluted in viral diluent to the appropriate concentration (10 μg/mL-0.01 μg/mL). Diluted sdAbs, as well as viral diluent (no sdAb) controls, were then mixed with an equal volume of the respective virus at 1,000 PFU/ml. Following a 1.5 h incubation at 37°C, sdAb/virus mixtures were then added to confluent monolayers of Vero cells. After a 1 h incubation period, an overlay containing 1.5% methylcellulose was added. Plaques were visualized following formalin fixation and staining with crystal violet 3 days post-infection. The number of plaques in the wells without sdAbs were counted and taken as control. The average number of plaques from replicates were then expressed as a percentage of that in the control wells.

### Cloning the donor construct: AE_CA6CC3

**Figure 2:**
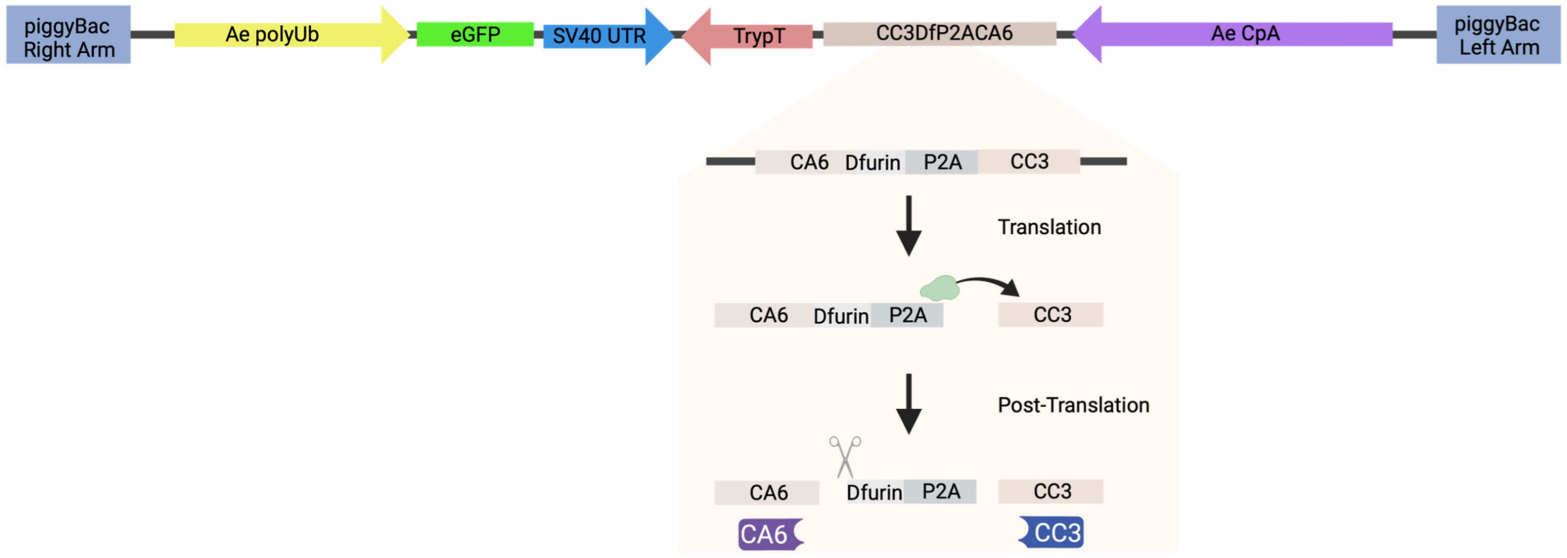
Schematic of construct design. AE_CA6CC3 construct design indicating each component of the transformation construct. AE_CA6CC3 was designed to express an eGFP transformation marker (green), constitutively expressed via the *Ae. aegypti* polyubiquitin promoter (Ae polyUb; yellow) to aid in screening and selection of transgenic mosquitoes. Additionally, a fusion peptide consisting of CA6 (i.e., sdAb or VHH), Dfurin 1, P2A, and CC3 (i.e., sdAb or VHH) (beige) was designed under the control of the *Ae. aegypti* carboxypeptidase A promoter (AeCpA; purple) to provide bloodmeal-upregulated, gut-specific gene expression. The P2A linker and Dfurin1 sequences were inserted between the sdAbs so that during translation, ribosomal skipping/translocation is induced (indicated by green ribosome and horizontal arrow) resulting in co-translational cleavage of the polyprotein. Post-translational processing by Dfurin 1 then removes the residual P2A sequence from the upstream gene (CA6; indicated by scissor icon) thus resulting in the production of individual CA6 (purple) and CC3 (blue) sdAbs. AE_CA6CC3 sequence is available through GenBank (accession: OQ921683).

To generate the piggyBac transposon-based plasmid that co-expresses both sdAbs (CA6 and CC3), herein referred to as “AE_CA6CC3,” a single coding sequence was designed *in silico* that contained the *Dfurin1* gene of *Drosophila melanogaster* Meigen (Diptera: Drosophilidae) cleavage site (R-Q-K-R) (Cano-Monreal et al., 2010) and *Porcine teschovirus*-1 2A (P2A) self-cleaving peptide (Liu et al., 2017) inserted between the CA6 and CC3 sequences (Figure 2). Sequences for these sdAb can be found in reference [26] (Liu et al., 2019). The entire transcript was codon-optimized for *Drosophila* and commercially synthesized by GENEWIZ, Inc. (South Plainfield, NJ). We then cloned the gene synthesis product containing the *Ae. aegypti* carboxypeptidase A (CpA) promoter, Dfurin 1, P2A, and the sdAb sequences into piggyBac [polyUb GFP] via GeneArt Gibson Assembly cloning (ThermoFisher Scientific, Waltham, MA) per manufacturer’s protocol. We sequence-validated the final AE_CA6CC3 construct using Sanger sequencing. All primers for cloning, and subsequent Sanger sequencing, were designed using SnapGene (San Diego, CA) software and obtained from Integrated DNA Technologies. Inc. (IDT; Coralville, IA). AE_CA6CC3 sequence is available through GenBank (accession: OQ921683).

### Mosquito rearing

*Ae. aegypti* Liverpool (LVP) eggs were obtained from BEI resources (Manassas, VA). Mosquitoes were reared and housed at 26°C, relative humidity 60–80%, and 12/12 h light/dark photoperiod. Larvae were fed Nishikoi fish food (Essex, England) and adult mosquitoes were provided with a 10% sucrose solution administered through cotton balls. Mosquitoes were provided defibrinated sheep’s blood (Colorado Serum Company; Denver, CO) using artificial membrane feeders for all bloodmeals.

### Mosquito transformations

Authors acquired necessary permissions prior to the start of the mosquito experiments. All studies were approved under IBC protocol (18–084) and conducted within an Arthropod Containment Level-3 (ACL-3) facility.

All transformations were carried out by adapting protocols previously described (Chen et al., 2021). PiggyBac donor plasmid (300 ng/μl; piggyBac [AE_CA6CC3]) that contains an enhanced green fluorescent protein (eGFP) transformation marker driven by the *Ae. aegypti* polyubiquitin promoter was co-injected with an *in vitro* transcribed piggyBac mRNA (300 ng/μl) into less than 1 h old embryos of *Ae. aegypti* LVP. The piggyBac-hsp70-transposase plasmid (Handler et al., 1998) was used as a template for *in vitro* transcription using the mMessage mMachine T7 Ultra kit (Thermofisher), followed by MEGAclear (Thermofisher) column purification. Surviving G_0_ females were mated to LVP males in pools of 20–25 mosquitoes. Each G_0_ male was mated individually with five LVP females in individual cages to isolate each independent line. G_1_ larvae were screened for green fluorescence using a Leica M165 FC fluorescence microscope. Positive G_1_ individuals were out-crossed to Liverpool mosquitoes to ensure that all transgene cassettes were stably inherited to the G_2_ generation.

### Western blots

Midguts from WT, AE1, and AE5 mosquitoes (*n* = 30/group; >G_3_ generation) were dissected 16 h after a bloodmeal and protein was extracted with ice-cold radioimmunoprecipitation assay buffer (RIPA buffer; 50 mM Tris-HCl pH 7.4, 150 mM NaCl, 0.25% Na-deoxy- cholate, 1% NP-40, 1 mM EDTA) containing one cOmplete^™^, Mini, EDTA-free Protease Inhibitor Cocktail tablet (MilliporeSigma, Burlington, MA). The entire sample was then homogenized and centrifuged at 4°C. Clarified supernatants were transferred to a fresh microcentrifuge tube, pulse-sonicated twice for 10 s each, and mixed with SDS loading buffer. All samples were then placed in a heating block at ~90–95°C for 10 min. Samples were run on a 4–20% SDS-PAGE and transferred to a nitrocellulose membrane. The membranes were then stained with Ponceau S stain to visualize protein transfer. Following destaining, membranes were probed with AffiniPure Goat Anti-Alpaca IgG, VHH domain primary antibody (128–005-232; Jackson ImmunoResearch, West Grove, PA) at a concentration of 15 μg/mL overnight at 4°C. The secondary antibody used was rabbit anti-goat HRP (HAF017; R&D Systems, Minneapolis, MN) at a dilution of 1:1000 per the manufacturer’s recommendation. Images were generated by applying Prometheus^™^ ProSignal^™^ Pico chemiluminescent ECL reagents (20-300B, Genesee Scientific, San Diego, CA) to the blots and visualized using an Azure c400 gel imaging system (Azure Biosystems, Inc., Dublin, CA).

### Vector competence experiments and virus titrations

Vector competence experiments were carried out by adapting protocols previously described (Bates et al., 2021). Female mosquitoes 5–7 days old (*n* = 50) were separated into cartons and sucrose starved for 16 h and H_2_O starved for 12 h prior to infection to promote blood-feeding. Mosquitoes were fed virus-spiked bloodmeals containing defibrinated sheep’s blood (Colorado Serum Company; Denver, CO) using artificial membrane feeders, and only fully engorged mosquitoes were separated into new containers. Pre- and post-infection blood samples were collected for use as back-titer calculations. Seven days post-infection, mosquitoes were cold-anesthetized and midguts and legs/wings were collected in viral diluent. Saliva was collected through forced salivation for 30 min in immersion oil and then mixed with viral diluent. Samples were stored at −80°C until analyzed by plaque assay. For virus titrations, all virus-negative mosquito samples were given a value of half the limit of detection (LOD/2) for statistical analyses.

### Statistics

For vector competence experiments, data were analyzed via a two-tailed Fisher’s exact test. For virus titration experiments, data were analyzed by Kruskal Wallis test with multiple comparisons with undetectable mosquito samples given a value of half the limit of detection (LOD/2) for statistical analyses.

## Results

### CA6 and CC3 display potent virus neutralization *in vitro*

**Figure 3:**
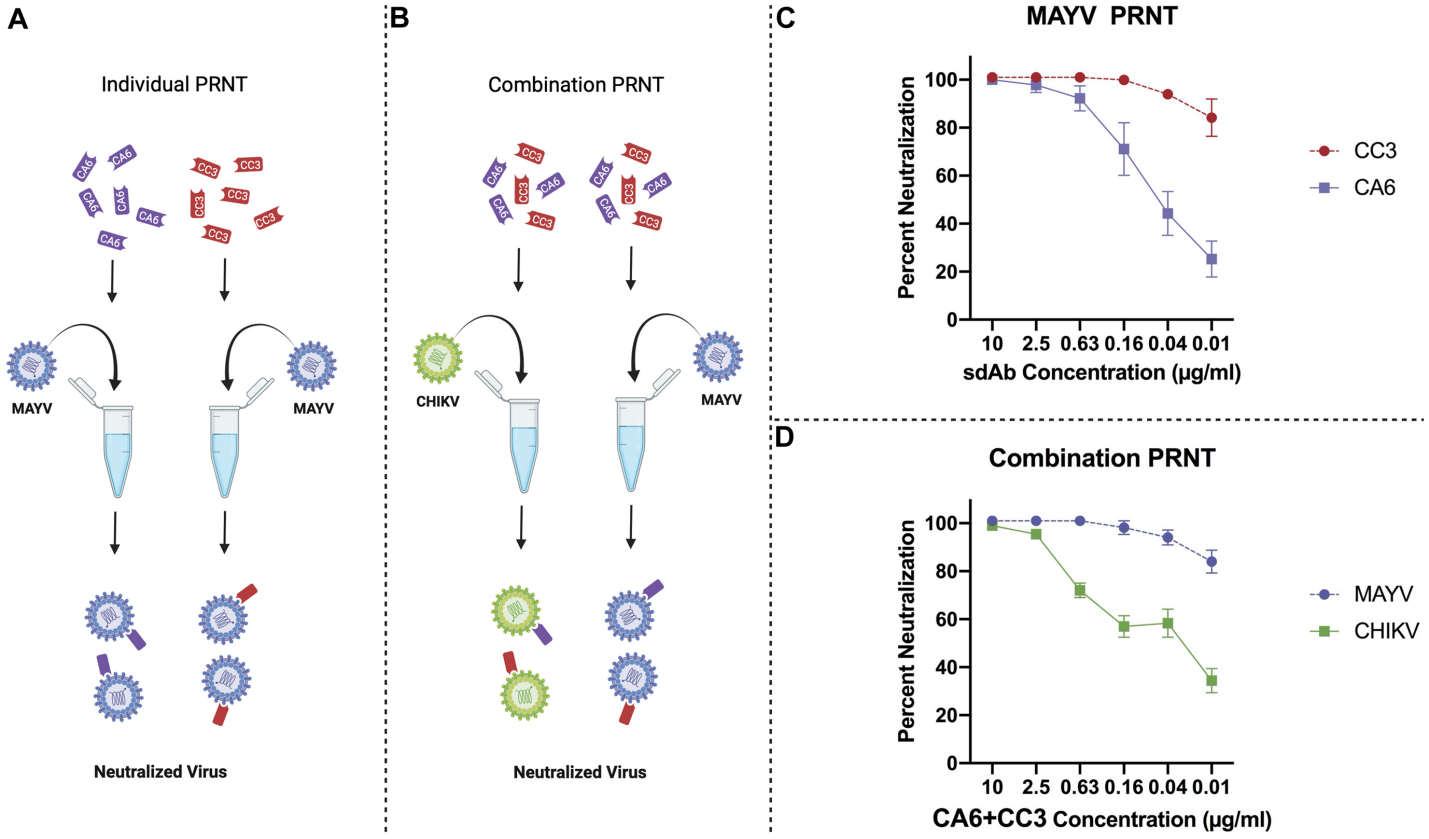
General schematic of PRNT methods and virus neutralization data. **(A)** Schematic representing PRNT method of MAYV neutralization using CA6 (dark purple) and CC3 (red). **(B)** Schematic representing combination PRNT method of CHIKV (green) and MAYV (light purple) neutralization via samples mixed with both CA6 (dark purple) and CC3 (red). **(C)** PRNT data provides percent neutralization of MAYV by CC3 (red, circles, dashed line) and CA6 (dark purple, squares, solid line). **(D)** Combination PRNT data provides percent neutralization of MAYV (purple, circles, dashed line) and CHIKV (green, squares, solid line) by mixed samples of both CA6 and CC3. Purified sdAb samples were serially diluted in viral diluent to the appropriate concentration (10 μg/mL–0.01 μg/mL) and then mixed with an equal volume of the respective virus at 1,000 PFU/ml. Data points are representative of six replicates obtained in two independent experiments. Error bars represent SD.

We previously showed that two anti-CHIKV sdAbs, clones CC3 and CA6, were potent at neutralizing CHIKV (Liu et al., 2019), and that CC3 also has strong neutralizing capacity against MAYV and other related alphaviruses (Liu et al., 2021). We then performed individual sdAb plaque reduction neutralization tests (Figure 3A; PRNTs) and combination PRNTs (Figure 3B). The individual PRNTs were performed to further validate CC3’s neutralization activity against MAYV and to demonstrate that CA6 has anti-MAYV activity (Figure 3C). We hypothesized that using both neutralizing sdAbs simultaneously would not interfere with neutralization capacity, thus we performed a series of combination PRNTs to evaluate the level of CHIKV and MAYV neutralization in the presence of both CC3 and CA6 (Figure 3D). These data demonstrate that the use of both CC3 and CA6 at concentrations greater than 0.16 μg/mL, maintain potent neutralization of both CHIKV and MAYV.

### Generation of transgenic *Ae. aegypti* colonies expressing sdAbs

**Figure 4:**
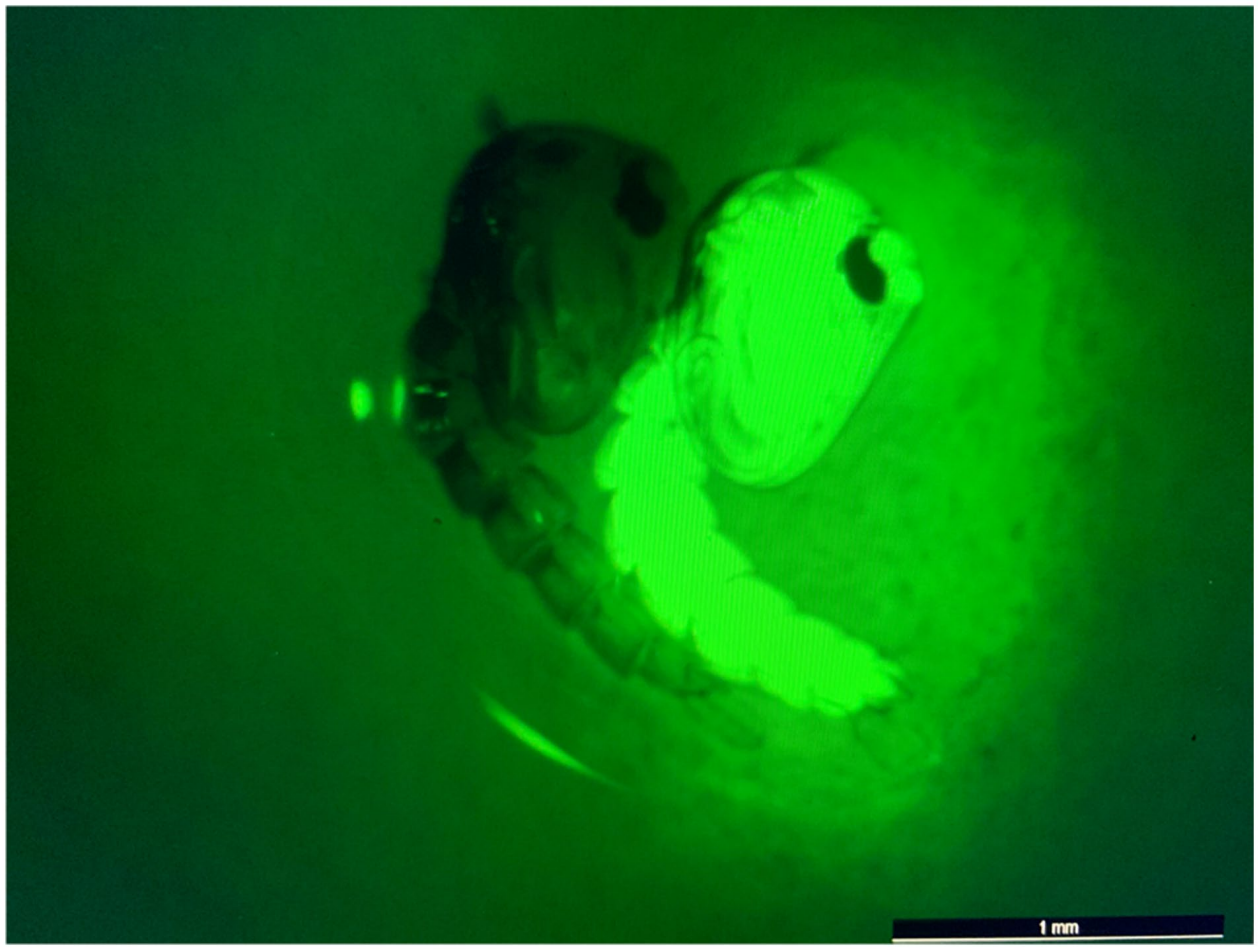
eGFP screening process of transgenic pupae. Image of non-transgenic (*left*) and transgenic/eGFP-expressing (*right*) pupae. eGFP screening was performed using a Leica M165 FC fluorescence microscope. All non-transgenic siblings were discarded of and not included in subsequent experiments.

We next constructed a piggyBac transposon-based plasmid, herein referred to as “AE_CA6CC3,” to co-express both sdAbs in *Ae. aegypti* mosquitoes. AE_CA6CC3 was designed to express both CC3 and CA6 under the control of the *Ae. aegypti* carboxypeptidase A promoter (AeCpA) (Moreira et al., 2000) to provide bloodmeal-upregulated, gut-specific gene expression (Figure 2). The eGFP transformation marker, constitutively expressed via the *Ae. aegypti* polyubiquitin promoter (Ae polyUb) (Anderson et al., 2010), was also included in the design to aid in screening and selection of construct-positive mosquitoes (Figure 4). To generate transgenic mosquitoes, the AE_CA6CC3 donor vector and *piggyBac* mRNA were co-injected into *Ae. aegypti* embryos and the resulting G_0_ individuals were outcrossed to wild-type (WT) mosquitoes. In the G_1_, GFP-positive individuals identified from each cross in were crossed with WT mosquitoes of the opposite sex to establish multiple independent lines (*n* = 5). Upon further observation, one of the transgenic lines was removed from experimentation due to an apparent male-linked insertion of our construct. The resulting four transgenic lines (AE1, AE2, AE4, and AE5) were reared for multiple generations to expand the colonies for preliminary vector competence studies.

### Preliminary analyses allow for selection of optimal transgene phenotype in two transgenic lines

We first performed preliminary vector competence experiments to determine which virus strains should be used to evaluate the transgenic lines. We compared two strains of CHIKV (CHIKV H-20235 and CHIKV SL-15649) and two strains of MAYV (MAYV 12A and MAYV TRVL-4675) in WT *Ae. aegypti* LVP mosquitoes. We spiked bloodmeals with each virus and evaluated virus infection, dissemination, and transmission 7 days post-infection (dpi). Our results indicated the use of CHIKV H-20235 and MAYV 12A for subsequent studies due to the high transmission rates (83 and 75%, respectively; Table 1) in WT mosquitoes. Additionally, both CHIKV H-20235 (Accession: KX262991) and MAYV 12A (Accession: KP842796) were isolated within the last 15 years.

**Table 1:** Preliminary screening of vector competence of WT *Ae. aegypti* LVP mosquitoes for CHIKV and MAYV strains.

Virus	IBM titer	Infection (midgut)	Dissemination (legs/wings)	Transmission (saliva)
CHIKV H-20235 (Asian)	8.53E+07	12/12 (100%)	12/12 (100%)	10/12 (83%)
CHIKV SL-15649 (ECSA)	4.00E+07	11/12 (92%)	9/12 (75%)	3/12 (25%)
MAYV 12A (L)	9.67E+07	12/12 (100%)	12/12 (100%)	9/12 (75%)
MAYV TRVL-4675 (D)	1.40E+08	11/12 (92%)	11/12 (92%)	5/12 (42%)

CHIKV H-20235 (representing the Asian lineage), CHIKV SL-15649 (representing the ECSA lineage), MAYV 12A (representing the L genotype), and MAYV TRVL-4675 (representing the D genotype) were used in these studies. Data are presented as the number of virus positive/number tested with rates of infection (virus present in midgut tissues), dissemination (virus present in legs/wings tissues), and transmission (virus present in saliva samples) indicated by percentages in parentheses. IBM, infectious blood meal.

Following the selection of virus strains, the transgenic lines (*n* = 4) were subjected to a preliminary vector competence analysis to assess susceptibility to CHIKV and MAYV. Multiple lines were generated and analyzed because transgene integration is unpredictable when using transposon-based delivery systems and testing of multiple lines facilitates identification of lines with the optimal transgene expression phenotype(s) (Reid et al., 2021). Female mosquitoes from the WT colony and each transgenic line were exposed to either CHIKV or MAYV viremic bloodmeals and infection, dissemination, and transmission rates were calculated 7 days later. AE1 and AE5 were selected for further experimentation since transmission rates were the lowest for each virus (Table 2). Specifically, compared to WT mosquitoes, AE1 transmission was reduced for CHIKV and MAYV (*p* = 0.002 and *p* = 0.004, respectively) and AE5 had a significant reduction (*p* = 0.008) in transmission for CHIKV and a near significant reduction (*p* = 0.056) for MAYV. Interestingly, we observed no differences in dissemination of CHIKV in our transgenic lines, which could be to lower overall systemic titers or due to leaky expression outside of the gut. Further studies can be conducted to assess expression in different compartments.

**Table 2:** Preliminary vector competence of transgenic and WT *Ae. aegypti* mosquitoes for CHIKV H-20235 and MAYV 12A.

Virus	Transgenic line	Infection (midgut)	Dissemination (legs/wings)	Transmission (saliva)
CHIKV H-20235 IBM titer: 6.23E+07	**AE1**	20/20 (100%) *1.000*	17/20 (85%) *0.231*	**2/20 (10%)** ***0.002***
	AE2	20/20 (100%) *1.000*	20/20 (100%) *1.000*	8/20 (40%) *0.343*
	AE4	20/20 (100%) *1.000*	19/20 (95%) *1.000*	9/20 (45%) *0.527*
	**AE5**	20/20 (100%) *1.000*	18/20 (90%) *0.487*	**3/20 (15%)** ***0.008***
	WT	20/20 (100%) *N/A*	20/20 (100%) *N/A*	12/20 (60%) *N/A*
MAYV 12A IBM titer: 9.87E+06	**AE1**	16/20 (80%) *0.106*	9/20 (45%) ***0.019***	**4/20 (20%)** ***0.004***
	AE2	20/20 (100%) *1.000*	20/20 (100%) *0.231*	14/20 (70%) *1.000*
	AE4	18/20 (90%) *0.487*	12/20 (60%) *0.155*	10/20 (50%) *0.333*
	**AE5**	18/20 (90%) *0.487*	**10/20 (50%)** ***0.041***	**7/20 (35%)** ***0.056***
	WT	20/20 (100%) *N/A*	17/20 (85%) *N/A*	14/20 (70%) *N/A*

Bold formatting indicates statistical significance. Data are presented as the number of virus-positive/number tested with rates of infection (virus present in midgut tissues), dissemination (virus present in legs/wings tissues), and transmission (virus present in saliva samples) indicated by percentages in parentheses. Two-tailed *p* values of infection, dissemination, and transmission (compared to WT) indicated in italics. Statistical analyses were performed using Fisher’s exact test in comparison to WT mosquitoes. IBM = infectious blood meal.

### Transgenic *Ae. aegypti* express CA6 and CC3

**Figure 5:**
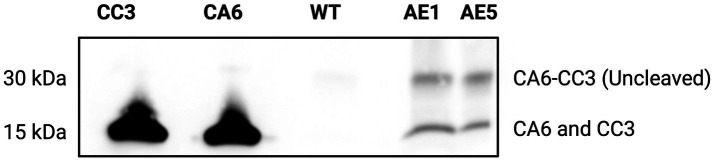
Western blot analysis to probe for the expression of sdAbs in AE1 and AE5. Western blots were carried out using homogenized midgut tissues from 16 h post-bloodmeal WT, AE1, and AE5 mosquitoes (*n* = 30/group). Purified CC3 and CA6 sdAbs (Liu et al., 2019) were used as controls and all samples were probed with AffiniPure Goat Anti-Alpaca IgG, VHH domain primary antibody. The presence of a 15 kDa band confirms the expression of the individual, cleaved sdAb proteins (CA6 and CC3) in transgenic, but not in WT, mosquito midgut samples. The presence of a 30 kDa band in the transgenic samples may indicate unsuccessful ribosomal skipping of the P2A linker sequence located between the CA6 and CC3 sdAbs, resulting in a (uncleaved) CA6-CC3 fusion protein.

To evaluate CA6 and CC3 transgene expression *in vivo*, transgenic *Ae. aegypti* mosquito lines AE1 and AE5 were subjected to Western blot analyses. Midguts from each transgenic line (G_3_ and greater) and WT mosquitoes were dissected 16 h post-bloodmeal. Purified CC3 and CA6 sdAbs (a kind gift from Dr. Ellen Goldman) were used as a positive control (Figure 5). As expected, sdAbs were expressed in the midgut tissues of mosquitoes from AE1 and AE5 but not WT mosquitoes. Notably, banding patterns which may suggest unsuccessful ribosomal-skipping of the P2A linker sequence (Liu et al., 2017) thus resulting in a fusion protein of the CA6 and CC3 sdAbs (Figure 5).

### AE1 and AE5 reduce overall transmission potential of both CHIKV and MAYV

With selection of the AE1 and AE5 transgenic lines, and evidence of successful sdAb expression in these lines, we performed a larger vector competence experiment to compare infection (virus present in midgut tissues), dissemination (virus present in legs/wings tissues), and transmission (virus present in saliva samples) rates to those of WT mosquitoes (Table 3). We observed a near-significant reduction in dissemination rate (*p* = 0.0547) and a significant reduction in transmission rate (*p* < 0.0001) for line AE1 with CHIKV. We also observed significant reductions in the dissemination and transmission rates for line AE5 (*p* = 0.0053 and *p* < 0.0001, respectively) with CHIKV. Interestingly, significant reductions in all rates were found with both AE1 and AE5 lines for the MAYV vector competence experiments (Table 3).

**Table 3:** Vector competence of transgenic and WT *Ae. aegypti* mosquitoes for CHIKV H-20235 and MAYV 12A.

Virus	Transgenic line	Infection (midgut)	Dissemination (legs/wings)	Transmission (saliva)
CHIKV H-20235 IBM titer: 5.63E+07	AE1	39/40 (97.5%) *1.0000*	35/40 (87.5%) *0.0547*	7/40 (17.5%) ***<0.0001***
	AE5	38/40 (95.0%) *0.4937*	32/40 (80.0%) ***0.0053***	10/40 (25.0%) ***<0.0001***
	WT	40/40 (100%) *N/A*	40/40 (100%) *N/A*	33/40 (82.5%) *N/A*
MAYV 12A IBM titer: 3.63E+07	AE1	29/40 (72.5%) ***0.0004***	23/40 (57.5%) ***0.0005***	16/40 (40.0%) ***0.0243***
	AE5	28/40 (70.0%) ***0.0002***	28/40 (70.0%) ***0.0198***	12/40 (30%) ***0.0016***
	WT	40/40 (100%) *N/A*	37/40 (92.5%) *N/A*	27/40 (67.5%) *N/A*

Bold formatting indicates statistical significance. Data are presented as the number of virus-positive/number tested with rates of infection (virus present in midgut tissues), dissemination (virus present in legs/wings tissues), and transmission (virus present in saliva samples) indicated by percentages in parentheses. Two-tailed *p* values of infection, dissemination, and transmission (compared to WT) indicated in italics. Statistical analyses were performed using Fisher’s exact test in comparison to WT mosquitoes. IBM, infectious blood meal.

**Figure 6:**
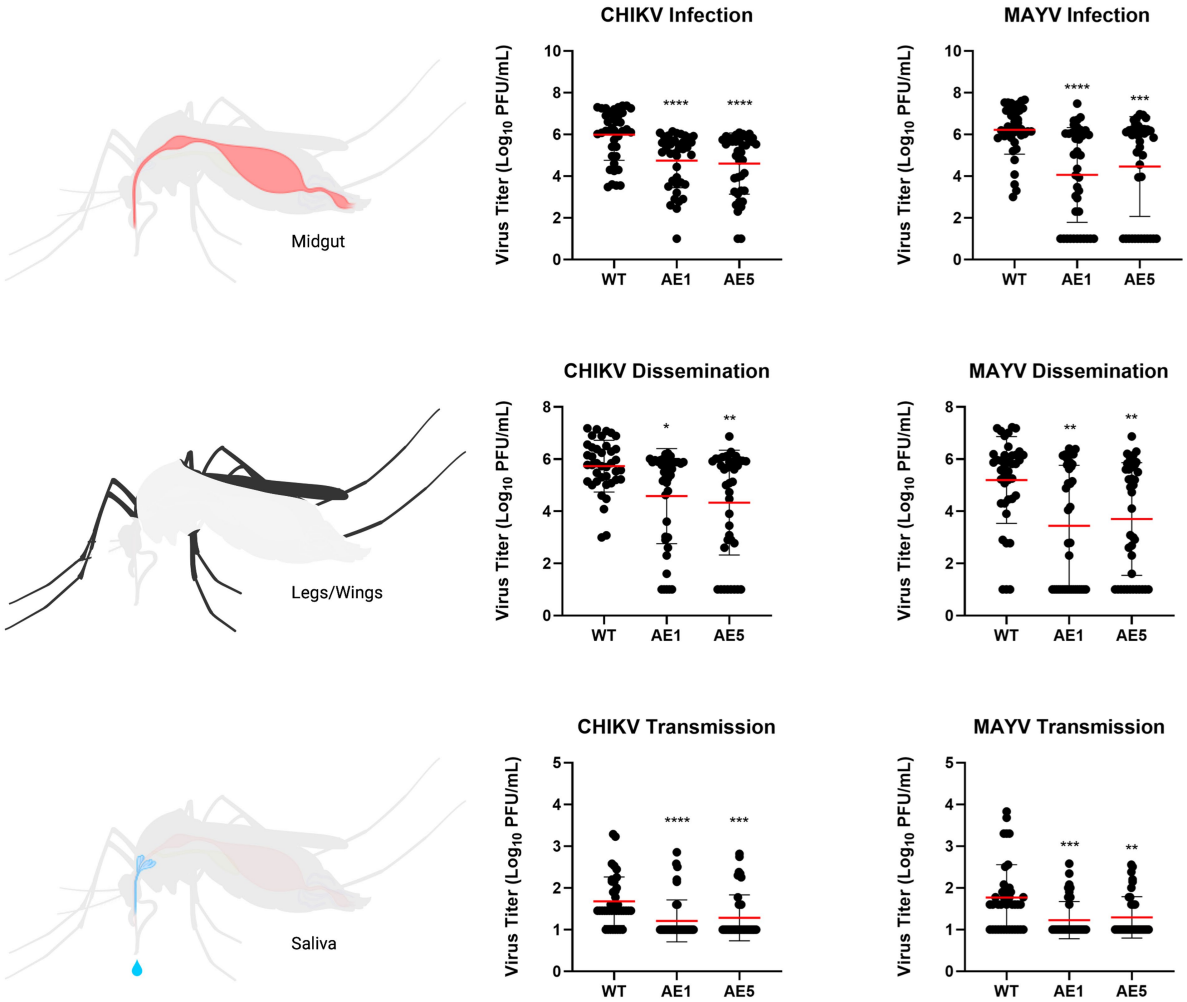
Plots of viral titers for CHIKV and MAYV in WT and transgenic mosquitoes. To evaluate the replication of CHIKV and MAYV, we measured viral titers infection (virus present in midgut tissues), dissemination (virus present in legs/wings tissues), and transmission (virus present in saliva samples). Female mosquitoes 5–7 days old were fed virus-spiked bloodmeals using artificial membrane feeders, and only fully engorged mosquitoes were separated into new containers. Seven days post-infection, mosquitoes were cold-anesthetized and individual samples were collected. Viral titers were measured via plaque assay on Vero cells 7 dpi. Data points of the 40 replicates obtained in two independent experiments are depicted on the plot. All undetected mosquito samples were given a value of half the limit of detection (LOD/2) for statistical analyses. Red horizontal bars represent the mean with SD. *p < 0.05, **p < 0.01, ***p < 0.001, ****p < 0.0001, Kruskal-Wallis test with Dunn’s correction for multiple comparisons. Infectious blood meal (IBM) titers: CHIKV = 5.63E+07, MAYV = 3.63E+07.

As mentioned, we did not see as strong of a reduction in dissemination of CHIKV in our transgenic lines and believed this could be due to lower overall systemic titers within the transgenic mosquitoes. Because of this, we sought to assess the replication of CHIKV and MAYV in these transgene-positive lines by measuring the viral titers present in each of the mosquito groups. We found significant reductions of CHIKV titers in AE1 and AE5 in infection (AE1 *p* < 0.0001; AE5 *p* < 0.0001), dissemination (AE1 *p* = 0.016; AE5 *p* = 0.008), and transmission (AE1 *p* < 0.0001; AE5 *p* < 0.0001) titers when compared to WT (Figure 6). Similarly, we observed significant reductions in MAYV replication in infection (AE1 *p* < 0.0001; AE5 *p* = 0.0008), dissemination (AE1 *p* = 0.0008; AE5 *p* = 0.0025), and transmission (AE1 *p* = 0.0003; AE5 *p* = 0.0024) (Figure 6). Alongside these data, we observed significant reductions in dissemination and transmission rates of infected transgenic mosquitoes when compared to WT mosquitoes (Supplementary Table S1). Altogether, these data demonstrate that both lines expressing sdAbs are more refractory to CHIKV and MAYV than WT mosquitoes.

## Discussion

CHIKV and MAYV have become increasingly prevalent in tropical regions of the world within the last few decades, highlighting a crucial need for novel approaches to control the spread of these crippling pathogens. Since 2004, CHIKV has been reported in over 100 countries (Silva et al., 2018; Vairo et al., 2019) and MAYV has the potential to spread similarly. In conjunction with sporadic outbreaks throughout the sub-tropical regions of the Americas, increased global travel to these endemic areas has resulted in imported cases of MAYV, as well as imported cases and autochthonous transmission of CHIKV, within the United States and Europe (Fischer and Staples, 2014; Bocanegra et al., 2016; Gossner et al., 2020). The overall goal of this study was to conceptualize a single control strategy that co-targets these emerging alphaviruses.

Our results demonstrate that the sdAbs, CC3 and CA6, have high neutralizing activity against both CHIKV and MAYV *in vitro* as demonstrated in our initial PRNT experiments (Figure 3). Furthermore, we successfully constructed and cloned a transformation construct (Figure 2) that was used to create several transgenic lines expressing eGFP (Figure 4). Of these transgenic lines, mosquito midgut samples from the AE1 and AE5 transgenic lines were subjected to Western blotting to verify expression of both CA6 and CC3 sdAbs (Figure 5). Interestingly, Western blot analyses revealed a dual banding pattern at ~30 kDa and ~15 kDa which may suggest unsuccessful ribosomal-skipping of the P2A linker sequence thus resulting in a fusion protein of the CA6 and CC3 sdAbs (Liu et al., 2017). Additionally, preliminary vector competence experiments using WT *Ae. aegypti* LVP revealed suitable virus strains of CHIKV and MAYV to be used in the transgenic vector competence experiments (Table 1). Each of the generated transgenic lines were then subjected to another preliminary vector competence experiment to determine which lines had the most refraction to CHIKV and MAYV and these results indicated the AE1 and AE5 transgenic lines were the most refractory to both viruses (Table 2). Thus, we performed a final, larger-scale, vector competence experiment to measure the changes in infection, dissemination, and transmission rates within the AE1 and AE5 lines when compared to WT mosquitoes. This vector competence experiment resulted in statistically significant reductions in dissemination and transmission rates for both lines with MAYV and for the AE5 line with CHIKV (Table 3). The AE1 line resulted in a statistically significant reduction of transmission for CHIKV and a near-significant (*p* = 0.0547) reduction in dissemination. Finally, we compared the overall group viral titers for each line and virus to that of WT titers and found statistically significant reductions of viral titers in each of the lines for both CHIKV and MAYV at the infection, dissemination, and transmission stages (Figure 6). To this end, we have generated transgenic mosquitoes that have refractory activity to both viruses and have therefore likely reduced transmission potential for CHIKV and MAYV in our transgenic mosquitoes.

Researchers have recently exploited genetic manipulation in problematic mosquito species as promising control strategies. Specifically, groups have generated mosquitoes expressing small RNAs or antibody fragments (e.g., scFv) to provide resistance to ZIKV and DENV (Yen et al., 2018; Buchman et al., 2019, 2020); however, our approach is unique based on the use of two camelid-derived sdAbs within a single vector to target two distinct alphaviruses. Importantly, our transgenic lines significantly reduced both CHIKV and MAYV replication and infection, dissemination, and transmission levels. This is particularly noteworthy because these viruses co-circulate throughout South America (Nunes et al., 2015; Vieira et al., 2015; Silva et al., 2018; Lorenz et al., 2019; Ganjian and Riviere-Cinnamond, 2020); thus, this strategy could limit the spread of both pathogens simultaneously.

While these data are promising, future studies are needed to determine the potential for these viruses to escape from sdAb-mediated neutralization within the transgenic mosquitoes. We hypothesized that expressing two individual sdAbs would reduce the probability for these viruses to generate antibody escape variants; however, this hypothesis needs to be tested in future studies. It should be noted that research from previous scFv studies have not indicated the development and/or selection for viral escape mutants (Reid et al., 2021). In addition, the stability of sdAb expression by these transgenic mosquitoes should also be assessed. As environmental factors such as temperature and mosquito diets affect *Wolbachia* density in transinfected mosquitoes (Ross et al., 2019), there could be similar effects on sdAb expression in our transgenic mosquitoes. Alongside the issues of transgene stability, fitness costs to the transgenic mosquitoes need to be evaluated. However, the stability of our transgene and any potential fitness costs could be circumvented by using this concept in combination with a gene-drive system. Gene-drive systems result in super-Mendelian inheritance and thereby greatly increase the stability of the transgene and can lead to overcoming any fitness costs resulting from the transgene (Macias et al., 2017). Future studies should also consider whether these mosquitoes are refractory to more distantly related alphaviruses, since our previous data has shown substantial cross-neutralization by the CC3 sdAb to a range of alphaviruses (Liu et al., 2021). In addition to these studies, determining the effect this reduction in vector competence has on the vectorial capacity of these transgenic mosquitoes would be crucial in support for this strategy. Vectorial capacity is defined quantitatively and is influenced by variables such as vector density, longevity, vector competence, and extrinsic incubation period. Further studies focusing on the significance of this change in vector competence as well as time-course studies evaluating any shifts in extrinsic incubation period are needed to evaluate the true potential of this concept to reduce transmission in a real-life setting. It should also be noted that the viral titers used in this study may be less than those found in nature as CHIKV viremia can reach high levels (~10^9^ copies/ml) during the actue phase of infection (Schwartz and Albert, 2010); however, studies have shown titers as low as ~10^4–5^ PFU/ml is adequate for mosquitoes to become infected on monkeys (Turell et al., 1992). Future studies determining the effect a range of viral titers has on the refractory activity shown here should be performed.

Altogether, our data provide strong support for the effectiveness of using transgenic mosquitoes expressing virus-specific sdAbs to control alphavirus transmission. This study further highlights the ability to target multiple arboviruses simultaneously, and thus has the potential to significantly reduce disease burden of multiple pathogens using a single defense strategy.

## Data availability statement

The raw data supporting the conclusions of this article will be made available by the authors, without undue reservation.

## Author contributions

EMW, AC, ZT, SLP, and JW-L designed the experiments. EMW, AC, PR, and CC performed all experiments. EMW and JW-L analyzed all data. EMW and JW-L wrote the manuscript and all authors edited the manuscript.

## Funding

EMW is supported by a doctoral fellowship from the Institute for Critical Technology and Applied Science (ICTAS) at Virginia Tech.

## Conflict of interest

The authors declare that the research was conducted in the absence of any commercial or financial relationships that could be construed as a potential conflict of interest.

## Publisher’s note

All claims expressed in this article are solely those of the authors and do not necessarily represent those of their affiliated organizations, or those of the publisher, the editors and the reviewers. Any product that may be evaluated in this article, or claim that may be made by its manufacturer, is not guaranteed or endorsed by the publisher.
