# Supplementary material for: Expression of anti-chikungunya single-domain antibodies in transgenic Aedes aegypti reduces vector competence for chikungunya virus and Mayaro virus
Source: Front Microbiol. 2023 Jun 12;14:1189176. doi: 10.3389/fmicb.2023.1189176 (PMC10291133; doi:10.3389/fmicb.2023.1189176)
Supplement: Supplementary file 1 [file Table_1.docx]

**Table S1.** Screening the vector competence of orally-infected transgenic (AE1 and AE5) and wild-type (WT) *Ae. aegypti* mosquitoes to CHIKV H-20235 and MAYV 12A. Percent infection (virus present in the midgut), disseminated of infected (virus present in legs/wings of those positive for infection), and transmitted of infected (virus present in saliva expectorant of those positive for infection) in transgenic mosquitoes were compared to the WT. Each group contained 40 mosquitoes. ***** *p*<0.05, two-tailed Fisher's Exact Test.

| **CHIKV** | | | **MAYV** | | |  |
| --- | --- | --- | --- | --- | --- | --- |
| Infected | Disseminated of infected | Transmitted of infected | Infected | Disseminated of infected | Transmitted of infected |  |
| **AE1** | 97.5 | 89.7 | 17.9* | 72.5* | 79.3 | 55.2 |
| **AE5** | 95.0 | 84.2* | 26.3* | 70.0* | 96.6 | 41.4* |
| **WT** | 100.0 | 100.0 | 82.5 | 100.0 | 92.5 | 67.5 |
